# Context influences conscious appraisal of cross situational statistical learning

**DOI:** 10.3389/fpsyg.2014.00691

**Published:** 2014-07-10

**Authors:** Timothy J. Poepsel, Daniel J. Weiss

**Affiliations:** Comparative Communication Laboratory, Department of Psychology and Program in Linguistics, The Pennsylvania State University, University ParkPA, USA

**Keywords:** cross situational statistical learning, contextual cues, mutual exclusivity, awareness, word learning

## Abstract

Previous research in cross-situational statistical learning has established that people can track statistical information across streams in order to map nonce words to their referent objects (Yu and Smith, 2007). Under some circumstances, learners are able to acquire multiple mappings for a single object (e.g., Yurovsky and Yu, 2008). Here we explore whether having a contextual cue associated with a new mapping may facilitate this process, or the conscious awareness of learning. Using a cross-situational statistical learning paradigm, in which learners could form both 1:1 and 2:1 word–object mappings over two phases of learning, we collected confidence ratings during familiarization and provided a retrospective test to gage learning. In Condition 1, there were no contextual cues to indicate a change in mappings (baseline). Conditions 2 and 3 added contextual cues (a change in speaker voice or explicit instructions, respectively) to the second familiarization phase to determine their effects on the trajectory of learning. While contextual cues did not facilitate acquisition of 2:1 mappings as assessed by retrospective measures, confidence ratings for these mappings were significantly higher in contextual cue conditions compared to the baseline condition with no cues. These results suggest that contextual cues corresponding to changes in the input may influence the conscious awareness of learning.

## INTRODUCTION

One of the difficulties faced by language learners is mapping words to objects. The word–world mapping problem poses a significant challenge for learners because there are often a near infinite number of possible objects that can be considered for a given word (Quine, 1960; Hart and Risley, 1995). How might learners overcome this mapping ambiguity? Many theories suggest that learners are constrained in the types of referents they will consider in a single learning environment. For example, the mutual exclusivity constraint suggests that learners prefer to assign a single label to an object. When children are presented with a familiar and unfamiliar object, they will assign a novel label to the unfamiliar object, since they already have a label for the familiar one (Markman and Wachtel, 1988). There are a host of other constraints that have been posited, such as the whole-object bias (Markman, 1991), the Principle of Contrast (Clark, 1983), social-pragmatic constraints (Clark, 1987; Tomasello and Barton, 1994; Baron-Cohen, 1997; Diesendruck and Markson, 2001), as well as linguistic cues (Gleitman, 1990), and the Novel-Name-Nameless-Category Principle (Golinkoff et al., 1992), among others.

In addition to the constraints that learners may bring to bear on this problem, it has recently been proposed that they may also employ a form of statistical learning across multiple learning environments to help overcome the challenge of indeterminacy. The underlying logic of this assertion is that word meanings may be ambiguous within the context of a single learning environment, but if learners aggregate information across multiple environments, then statistical information can help to disambiguate which words belong with which objects. This idea was modeled in a laboratory experiment by Yu and Smith (2007) who had adult participants view several sets of objects on a computer screen while hearing their labels played in random order. Given the randomized presentation of words, learners could not use any single presentation to identify which word belonged with which object. However, if learners could aggregate information across multiple scenes (since objects appeared several times in different contexts throughout familiarization), over time they would be able to identify which words cohered with which objects. In fact, both adults and children were successful in this cross-situational statistical learning task (hereafter CSSL; Yu and Smith, 2007; Smith and Yu, 2008; Kachergis et al., 2009; Fazly et al., 2010; Fitneva and Christiansen, 2011), suggesting that learners are capable both of tracking information across scenes in order to deduce correct mappings, and importantly, retaining these mappings at significant delay from training (Vlach and Sandhofer, 2014).

Since the initial studies, CSSL has been extended to investigate how learning may occur when there is not a perfect one-to-one correspondence between objects and their referents (Yurovsky and Yu, 2008; Ichinco et al., 2009; Kachergis et al., 2009; Poepsel et al., 2012). For example, Yurovsky and Yu (2008) investigated whether mutual exclusivity effects would emerge if learners first acquired a set of mappings between objects and labels, and then in the second half of familiarization experienced a new mapping for a subset of the objects (i.e., one word mapped to two objects). In this condition, learners were capable of overcoming mutual exclusivity and learned both the first and second referents. However, in a direct preference test between both the first and second referents, learners tended to demonstrate a primacy bias, preferring the initial mapping relative to the more recent mapping. In a subsequent experiment, learners were able to acquire two mappings for a word even when both object mappings were intermingled throughout the familiarization period (i.e., unlike the initial condition, there was no distinction between the first and second half of training). Notably, in a third experiment, Yurovsky and Yu (2008) reran both experiments and asked participants to provide confidence ratings after every trial in order to elicit a measure of conscious knowledge about their learning. Learners were more confident in the condition in which the first and second mapping were separated during familiarization. They were also more confident about the primacy mapping relative to the recency mapping, a finding that corresponded to the preference results for primacy in the separate familiarization condition but not in the mixed condition. Overall, this set of experiments found only weak evidence for a mutual exclusivity bias.

A follow up experiment by Ichinco et al. (2009) revisited whether learners are subject to mutual exclusivity constraints within the CSSL paradigm. In the first block of familiarization, participants learned one set of word object mappings, and then in a second familiarization, they learned new one-to-one mappings along with a set of transferred words (or objects) that had previously been learned. These transferred words and objects appeared in the context of new word–object mappings, but were perfectly correlated in their co-occurrence with one of these new word–object mappings such that a double mapping could be formed. Under these circumstances, learners favored mutual exclusivity, mastering the first (primacy) mappings, and ignoring the statistically valid second (recency) mappings. From this pattern of results, the authors argue against a simple associative account for CSSL, instead endorsing the intentional word-learning model (Frank et al., 2009). By contrast, Kachergis et al. (2009) view the different results evidenced in these two studies as a function of complex associative learning, with the Ichinco et al. (2009) study essentially finding mutual exclusivity due to a blocking effect. In their study, Kachergis et al. (2009) found that learners could adaptively ignore mutual exclusivity when the input was manipulated to provide greater evidence for a new mapping.

A study in our lab further investigated whether mutual exclusivity effects in CSSL could be attenuated, in this case by adding a contextual cue to the second familiarization (such as a change in speaker voice). Such effects are mirrored in real-word acquisition. For example, if two speakers produce different descriptions for a novel object there may be no penalty for online interpretation. However, if a single speaker produces both descriptions, there is a cost associated with violating the initial description (e.g., Metzing and Brennan, 2003; Trude and Brown-Schmidt, 2012). This finding is broadly consistent with experiments that have explored the role of contextual cues in statistical learning in the context of a speech segmentation task. Several studies have demonstrated that the addition of a contextual cue that corresponds with a change in structures facilitates the acquisition of multiple structures (e.g., Gebhart et al., 2009; Weiss et al., 2009; Mitchel and Weiss, 2010).

In our previous study, we extended the investigation of contextual cues to the domain of statistical word learning. In the first experiment, we replicated the results of Ichinco et al. (2009) using two distinct familiarization blocks, transferring six words learned in the first familiarization to a second familiarization in which they were remapped to new objects (Poepsel et al., 2012; Weiss et al., in preparation). We then extended this finding by presenting the first familiarization in one voice and the second familiarization in a new voice (with either the same or different accent). This change was sufficient to improve the learning of the new mapping available during the second block of familiarization. Likewise, explicitly informing participants that they would be able to remap words in the second familiarization (with the voice held constant) facilitated the formation of new mappings between previously learned words and new objects in the second familiarization. These data suggest that in the process of statistical learning, learners are sensitive to the context in which statistics occur. Learners appear to be capable of exploiting this contextual sensitivity in order to form multiple representations (evidenced in the CSSL paradigm by learning many-to-one mappings). These findings accord with the experience of learners in a bilingual environment who could benefit by relaxing or never developing the mutual exclusivity preference in order to acquire translation equivalents. Consistent with this idea, several studies have found mutual exclusivity is not a hard constraint for bilinguals (e.g., Byers-Heinlein and Werker, 2009; Houston-Price et al., 2010), while modeling results suggest that the development of mutual exclusivity is itself dependent on the type of input that learners receive (McMurray et al., 2012).

To date, studies of the role of contextual cues in statistical learning have striven to explore their effects using retrospective measures of learning that likely reflect implicit learning (Gebhart et al., 2009; Weiss et al., 2009; Mitchel and Weiss, 2010). While the consensus view is emerging that statistical learning and implicit learning are more similar than different (e.g., Cleeremans et al., 1998; Hunt and Aslin, 2001; Conway and Christiansen, 2005; Perruchet and Pacton, 2006), far fewer studies in the statistical learning domain have concerned themselves with the extent to which learning is accessible to conscious awareness (Franco et al., 2011). Particularly within the realm of word learning, it is natural to inquire whether learners are aware of the matches between objects and their potential referents. A few such efforts have recently been undertaken by means of tracking learner’s estimation of their knowledge states over the course of training (e.g., Yurovsky and Yu, 2008; Medina et al., 2011; Vlach and Sandhofer, 2014). The initial findings predominantly suggest that learners are aware of their knowledge of mappings in CSSL tasks. In the present study, we sought to extend research by determining whether contextual cues might exert an effect on the conscious appraisal of learning (i.e., learners’ explicit estimation of their knowledge state) in a statistical learning paradigm. To accomplish this, we extended our previous study of CSSL, combining the methods of previous studies of mutual exclusivity effects within this paradigm (i.e., Yurovsky and Yu, 2008; Ichinco et al., 2009). We presented learners with two stages of familiarization (similar to Ichinco et al., 2009). The first familiarization contained eighteen one-to-one mappings. In the second familiarization, we then transferred six learned words from the initial set and remapped them to new objects. In addition, we presented learners with twelve new one-to-one mappings. In addition to using retrospective measures of learning, we asked participants to rate their confidence in word–object mappings after each presentation during familiarization (similar to Yurovsky and Yu, 2008). In the first condition, we provided no indexical cues to distinguish between the first and second familiarization. In the second and third condition, we provided contextual cues in the form of a voice change (Condition 2) and an explicit set of instructions (Condition 3). We were interested in whether the presence of a contextual cue might attenuate any mutual exclusivity bias present during test. Further, we were interested in whether the presence of a contextual cue might alter how learners rated their confidence in mappings throughout familiarization.

## MATERIALS AND METHODS

### PARTICIPANTS

In Condition 1, 20 introductory Psychology students (15 female and 5 male; 18–23 years) participated for course credit. In Condition 2, another 20 introductory Psychology students (11 female and 9 male; 18–25 years) participated for course credit. None had participated in Condition 1. In Condition 3, 21 introductory Psychology students (13 female and 8 male; 18–22 years) participated for course credit. None had participated in Conditions 1 or 2. None of the subjects had any prior experience with statistical learning experiments. The data of one participant in Condition 3 were excluded due to experimenter error. Five additional participants (three in Condition 2 and two in Condition 3) failed to reach a criterion score in the test following the first familiarization phase (see below) and were subsequently dismissed from the experiment and excluded from the statistical analyses.

### STIMULI

**FIGURE 1:**
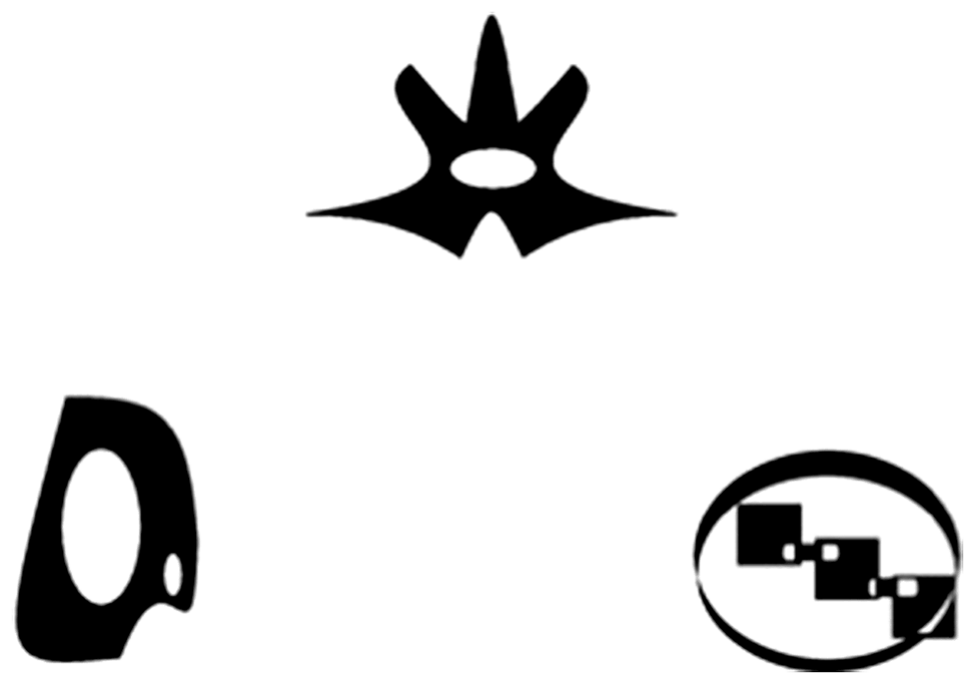
**An example of the visual array that participants saw in each familiarization trial**.

Stimuli consisted of a set of 54 unique word–object pairs created by randomly pairing novel objects with nonce words. Objects were black and white complex line drawings (see **Figure 1** for examples). Eight of these objects appeared in the stimuli used by Creel et al. (2008), and served as templates for the creation of the remaining 46, using MS Paint ©. All objects were converted to a jpeg file format with a size of 150 × 150 pixels.

Nonce words had American English phonological patterns and consisted of an equal distribution of monosyllabic, disyllabic, and trisyllabic items (e.g., chost, thecker, coronick) chosen from the english lexicon project (ELP) non-word database (http://elexicon.wustl.edu; see **Table 1** for a full listing of nonce words). Words chosen for this experiment were between four and ten characters in length, and based on data from the ELP had an average of 2.2 orthographic neighbors and a bigram mean of 2022. The words were rendered in both a female American English voice (*Crystal*) and a male American English voice (*Mike)* using the AT&T Natural Voices text-to-speech synthesizer (http://www.naturalvoices.att.com), and subsequently converted into WAV files sampled at 22050 Hz.

**Table 1:** Nonce words used in Experiments 1 and 2, organized by syllable count.

Monosyllabic	Bisyllabic	Trisyllabic
barsh	briskle	baturate
blep	crinklow	calorix
chost	dounger	caprion
crid	durrow	clamoreck
daint	grinter	coronick
drock	haser	haterfront
dulch	lattle	interlade
feech	masset	jatterside
frane	mubble	latercress
glack	murler	naureate
glink	pangle	overlood
gotch	patchet	perminal
plock	peadle	rentacle
plunt	pedline	tanderer
scown	pritter	thermistar
slute	tallot	todular
sunch	tarren	tonogram
veam	thecker	ventuker

All experiments were conducted in a sound-attenuated chamber and were programmed using E-Prime 2.0. Following completion of this task, participants filled out a language history questionnaire (LHQ), which contained questions about prior language learning experiences, demographic information, as well as effort spent on the experimental task.

### PROCEDURE

The experimental procedure was similar to that reported for experiments 2–4 in Poepsel et al. (2012). In the present study, participants completed two familiarization phases, each of which was followed by a test phase. During familiarization phases, 18 word–object pairs were presented over a series of 36 trials. A fixation cross appeared for 750 ms preceding each familiarization trial. Every trial consisted of three objects appearing simultaneously on a video monitor concurrent with the sequential presentation of three nonce words at 3 s intervals through noise-canceling headphones. Objects appeared in a fixed array in which two objects occupied the upper right and left areas of the screen and one object occupied the lower middle half of the screen. From trial to trial, object locations within this array as well as auditory word orders were randomly assigned, such that it was impossible to know which word corresponded with which object. The ordering of the trials was pseudo-randomized such that no word–object pair appeared in consecutive familiarization trials. Overall, each word–object pairing occurred six times during familiarization.

Immediately following each familiarization trial, participants were asked to judge how well they knew the name of each object. In a series of three presentations, participants viewed one of the objects from the preceding familiarization trial centered on the screen. Above the object was text which read, “Please rate how confident you are that you know this object’s name,” and below the object was a nine point scale, where “1” was marked as “Not Confident,” and 9 was marked as “Very Confident.” Participants rated their confidence by pressing the corresponding number on a keyboard, with no time limit for making a response. In these confidence-rating trials, the ordering of the objects from the preceding trial was randomized.

Following the first familiarization phase, participants completed a four-alternative forced-choice test (4AFC), in which chance performance was 25%. Each word–object pair was tested once for a total of 18 test trials. On each test trial, participants saw four objects and heard a single word. Three of these objects were distractors randomly selected from the set of objects presented within the familiarization phase. The remaining object was the correct referent for the presented word. The objects in a test trial were presented simultaneously, with one object located in each corner of the screen. Each object was labeled with a number (1–4). Participants were asked to press the number key corresponding to the correct referent of the word. There was no time limit for making a response.

In order to proceed to the second familiarization phase, participants had to achieve a minimum score of 10 correct responses (out of 18 total). This criterion was established in a previous study of mutual exclusivity effects in CSSL (Poepsel et al., 2012) in order to ensure that learners initially acquired the majority of mappings. Failure to achieve this criterion ended the experiment. As reported above, five participants failed to reach this criterion and were dismissed from the experiment prior to the second familiarization. The second familiarization phase also contained 18 word–object pairs. These consisted of a combination of novel word–object pairs and familiar words that received new object-mappings. Specifically, six learned words from the firstfamiliarization were transferred to the second familiarization. The set of transferred words consisted of the first six words a participant correctly mapped in the test following the first familiarization phase. Each transferred word was mapped to a novel object (i.e., an object unique to the second familiarization). The remaining 12 word–object pairs of the second familiarization consisted of entirely novel words and objects. All other properties of the second familiarization were identical to those of the first.

The test following the second familiarization also differed from the test that followed the first familiarization. This test consisted of 54 trials. The first 18 trials focused exclusively on the second familiarization and tested the set of 12 new word–object mappings as well as the set of six new remapped words from the first familiarization. The order of these trials was randomized. The next 18 trials retested the 1:1 mappings from the first familiarization. Note that this latter test was not an identical test to the one received after the first familiarization (the design was the same, but the pairings for the distractors differed). Following this, a set of six trials tested whether participants displayed a preference for the primacy or recency mappings of remapped words. On each preference trial, participants heard a transferred word and saw a visual array containing its first (primacy) and second (recency) familiarization object mappings, along with two distractor objects. A final set of six trials retested the 2:1 mappings from the second familiarization.

There were three conditions in this experiment. In Condition 1, all stimuli across the first and second familiarizations were presented in the same American English female voice (Voice 1). In Conditions 2 and 3 there was a contextual cue that differentiated between the first and second familiarization. In Condition 2, stimuli in the first familiarization were presented in female Voice 1 while stimuli in the second familiarization were presented in an American English male voice (Voice 2) whose fundamental frequency was, on average, 70 Hz lower than that of Voice 1. In Condition 3, the stimuli in both the first and second familiarization were presented in the same voice (Voice 1). However, there was an explicit contextual cue that was presented before the beginning of the second familiarization. Specifically, participants viewed a message that read: “In this part of the experiment, several of the words you have just learned will receive new object mappings.”

## RESULTS

**FIGURE 2:**
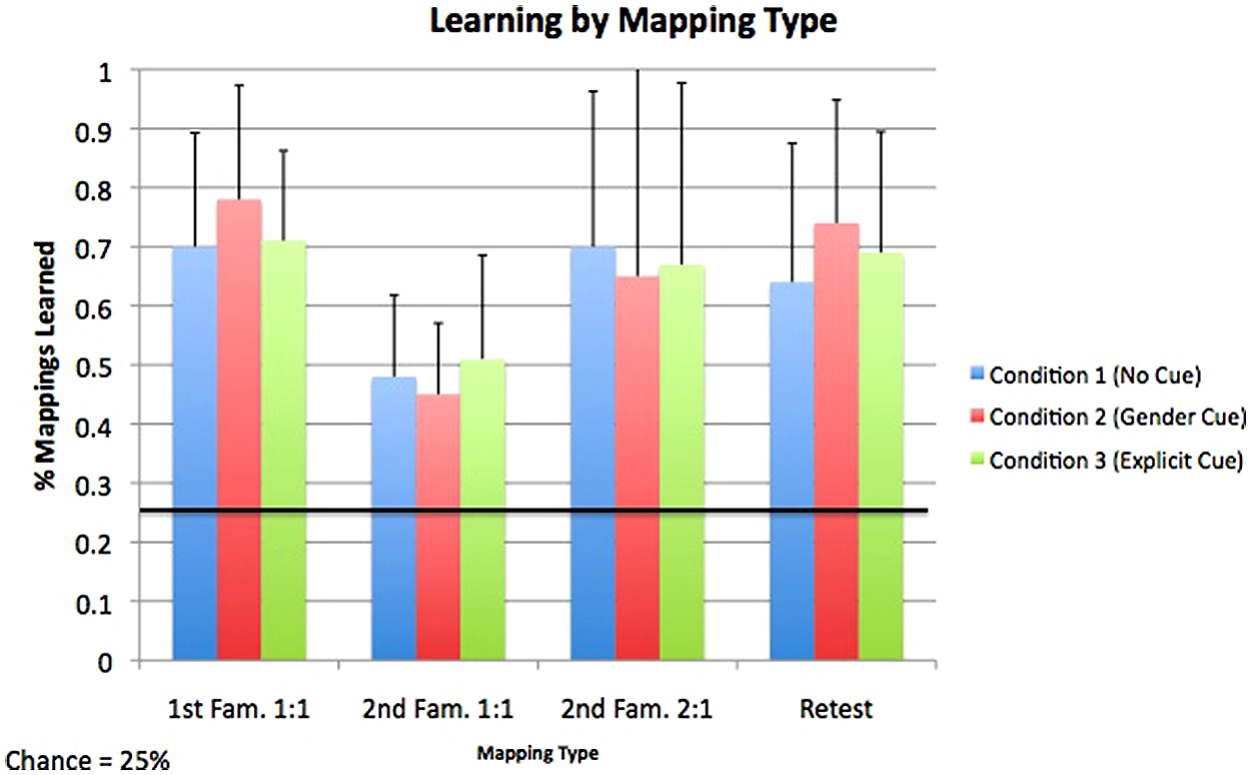
**Accuracy for each mapping type across each of the three experimental conditions.** For all mapping types and in all conditions, accuracy was above the level of chance, indicating successful acquisition of both 1:1 and 2:1 mappings. Error bars represent one SE.

All test items were 4AFC, and thus chance learning in the tests following the first and second familiarizations was set at 25%. Learning means for each condition and mapping type are shown in **Figure 2**. We used a 4 (Trial Type) × 3 (Condition) repeated measures ANOVA to investigate the factors that influenced accuracy at test. Trial type was a within-subjects factor, while Condition was a between subjects factor. There was a main effect of trial type [*F*(3,57) = 31.54, *p*< 0.001, η^2^ = 0.35], such that learning of 1:1 mappings in the second familiarization (*M* = 47.9%, SE = 1.9%) was significantly lower than learning of all other mapping types [i.e., first familiarization 1:1 mappings (*M* = 73.2%, SE = 2.3%), second familiarization 2:1 mappings (*M* = 67.2%, SE = 3.9%), Retest mappings (*M* = 68.8%, SE = 2.7%)] as shown by *post hoc* pairwise comparisons (all *p*s < 0.001). The interaction between Trial type and Condition was not significant [*F*(6,177) = 1.51, *p*= 0.18], nor was the between-subjects factor of Condition [*F*(2,59) = 0.12, *p*= 0.89], indicating that accuracy on each trial type did not vary between the conditions, and also that was there no overall difference in accuracy between conditions.

We compared accuracy on each test trial type against the level of chance (25%) in a series of single-sample *t*-tests. As no differences in accuracy within any test trial type were found between the conditions, results from all conditions were collapsed together. Learning exceeded chance for all test-trial types [first familiarization 1:1 mappings: *t*(60) = 21.1, *p*< 0.01; second familiarization 1:1 mappings: *t*(60) = 12.3, *p*< 0.01; Retest: *t*(60) = 15.9, *p*< 0.01; 2:1 mappings: *t*(60) = 10.8, *p*< 0.01], demonstrating that participants were able to successfully acquire both 1:1 and 2:1 mappings in all conditions.

Two one-way ANOVAs explored how performance on 1:1 mappings in the first familiarization compared to performance on 1:1 mappings in the second familiarization as well as on retest trials. As in previous comparisons, results from all three conditions were collapsed. There was a highly significant difference in performance between 1:1 mappings in the first familiarization (*M* = 73.3%, SD = 18.0%) and second familiarization [*M* = 47.9%, SD = 14.6%; *F*(1,119) = 74.4, *p*< 0.001, η^2^ = 0.38]. There was no significant difference in performance, however, between 1:1 mappings in the first familiarization and Retest mappings [*M* = 68.9%, SD = 21.6%; *F*(1,119) = 1.5, *p*= 0.22].

**FIGURE 3:**
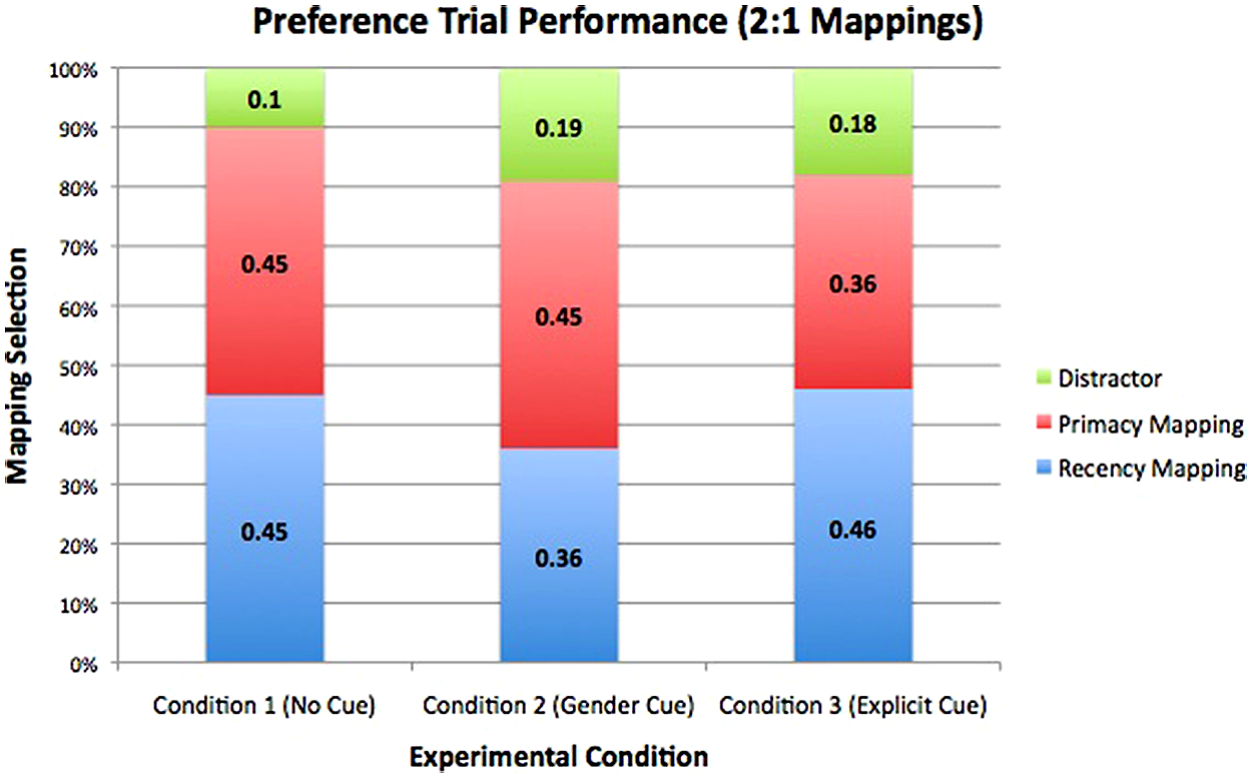
**Preference data collected from test trials in which participants saw both the primacy and recency mapping of a word, along with two distractor objects.** Within each of the three conditions, participants showed no significant preference for primacy or recency mappings.

In the test of learning following the second familiarization of the present experiment, participants encountered a set of trials that assessed whether participants showed a preference for primacy or recency mappings of transferred words. Within individual conditions, participants showed no significant preference for either the primacy or recency mapping of transferred words [Baseline: *t*(20) = 0.00, *p*= 1; Gender Cue: *t*(20) = -0.92, *p*= 0.37; Explicit Cue: *t*(20) = 0.98, *p*= 0.34] (see **Figure 3**).

We used a series of 2 (Contextual Cue) × 6 (Occurrence of Word–Object Pair) ANOVAs to investigate the factors that influenced confidence ratings during familiarizations. Separate ANOVAs, identical in design, were run for the set of 1:1 mappings from the first familiarization, 1:1 mappings from the second familiarization, and 2:1 mappings from the second familiarization. Contextual Cue was a between subjects factor, coded as 1 for conditions without a contextual cue (i.e., the baseline condition), and 2 for conditions with a contextual cue (i.e., the gender and explicit cue conditions). Occurrence of word–object pair was a within subjects factor with six levels, one for each of the six occurrences of a word–object pair that learners rated.

For 1:1 mappings in the first familiarization, there was a main effect of Occurrence of word–object pair [*F*(5,295) = 16.87, *p*< 0.001, η^2^ = 0.23], such that confidence ratings for word–object pairs rose significantly across the six presentations of each pair during training. The interaction between Occurrence and Context was also significant [*F*(5,295) = 2.46, *p*= 0.03, η^2^ = 0.04], suggesting that learners in the baseline condition gave higher estimates of their confidence in mappings over the earlier presentations of a word–object pair relative to those in the contextual cue condition, but lower estimates of confidence over the later presentations. Finally, the between subjects factor of Context did not reach significance [*F*(1,59) = 0.07, *p*= 0.79], suggesting that there was no overall difference in how learners rated their confidence in mappings between the baseline and contextual cue conditions for 1:1 mappings in the first familiarization.

For 1:1 mappings in the second familiarization, there was again a main effect of Occurrence of Word–Object Pair [*F*(5,295) = 91.3, *p*< 0.001, η^2^ = 0.62], such that confidence ratings for word–object pairs rose significantly across the six presentations of each pair during training (see **Table 2** for confidence rating means and SE by presentation). The interaction between Occurrence and Context was not significant [*F*(5,295) = 0.32, *p*= 0.9]. The between- subjects factor of Context again did not reach significance [*F*(1,59) = 0.01, *p* = 0.91], suggesting that there was no overall difference in how learners rated their confidence in mappings between the baseline and contextual cue conditions.

**Table 2:** Means and SE (in parentheses) for confidence ratings by mapping type and occurrence of a word–object pair within a familiarization.

Occurrence of word–object pair	First fam. 1:1 mappings	Second fam. 1:1 mappings	Second Fam. 2:1 mappings
First	4.8 *(0.27)*	3.0 *(0.26)*	3.0 *(0.27)*
Second	5.25 *(0.25)*	3.91 *(0.27)*	3.9 *(0.27)*
Third	5.41 *(0.27)*	4.64 *(0.3)*	4.75 *(0.27)*
Fourth	5.68 *(0.28)*	5.42 *(0.29)*	5.42 *(.028)*
Fifth	6.32 *(0.26)*	5.92 *(0.3)*	6.0 *(0.26)*
Sixth	6.51 *(0.26)*	6.40 *(0.3)*	6.66 *(0.28)*

**FIGURE 4:**
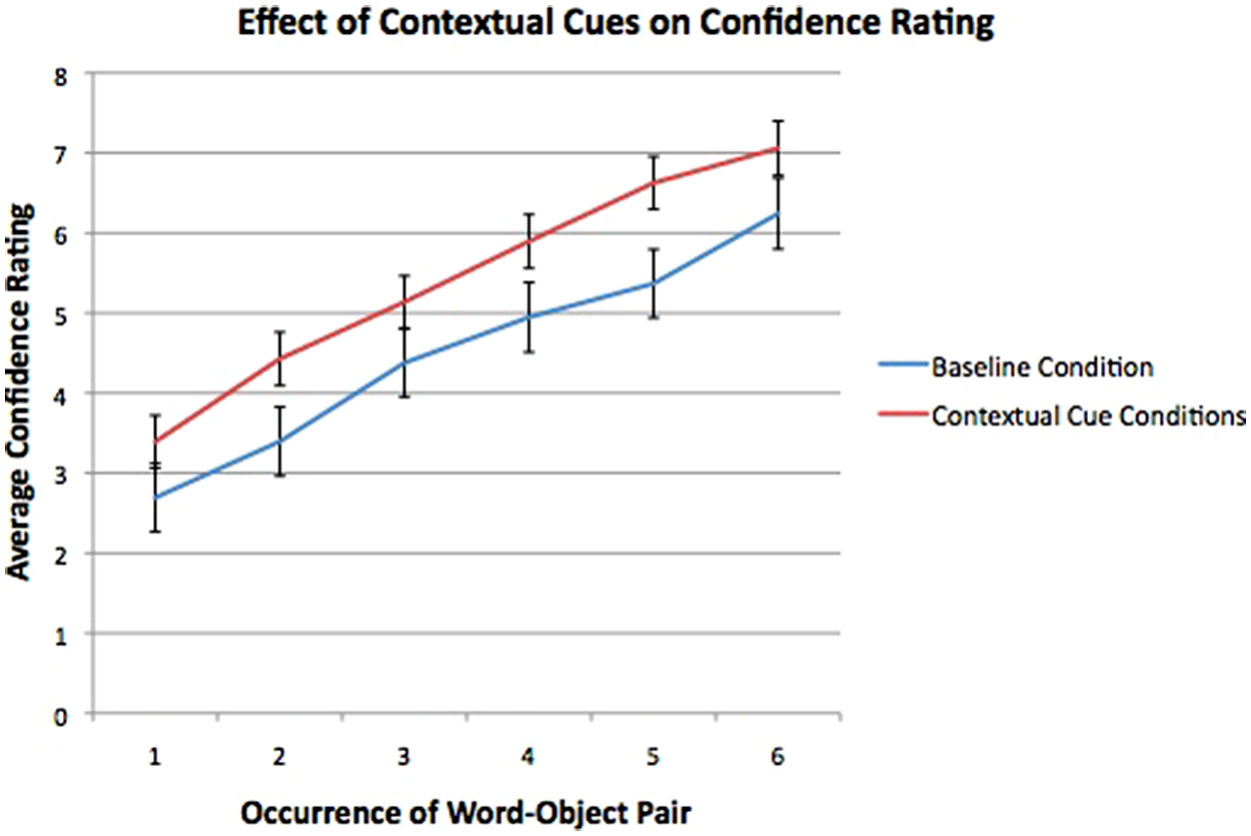
**Confidence ratings for 2:1 mappings in the second familiarization, with error bars representing one SEM.** Confidence ratings for 2:1 mappings in the two contextual cue conditions (combined here and shown in red) were significantly higher than those in the baseline condition (shown in blue).

For 2:1 mappings in the second familiarization, there was again a main effect of Occurrence of word–object Pair [*F*(5,295) = 65.8, *p*< 0.001, η^2^ = 0.57]. The interaction between Occurrence and Context was not significant [*F*(5,295) = 0.39, *p*= 0.86]. However, the between- subjects factor of Context for 2:1 mappings was significant [*F*(1,59) = 4.1, *p*= 0.04, η^2^ = 0.08], implying that ratings for 2:1 mappings in the contextual cue conditions were higher than those in the baseline condition (see **Figure 4**).

Finally, for each of the three mapping types (first familiarization 1:1, second familiarization 1:1, second familiarization 2:1) we examined the correlation between average accuracy at test and the average confidence rating for that mapping type. For 1:1 mappings in the first familiarization, we found a marginally significant positive correlation (*R* = 0.225, *p = 0*.08, *N* = 60) between accuracy and confidence ratings. For 1:1 mappings in the second familiarization, we found a significant positive correlation between accuracy and confidence ratings (*R* = 0.45, *p*< 0.001, *N* = 60). For 2:1 mappings in the second familiarization, we also found a significant positive correlation between accuracy and confidence ratings (*R* = 0.29, *p*= 0.04, *N* = 60). Thus, for all mapping types, we found a significant (or marginally significant) positive relationship between confidence ratings and accuracy.

## DISCUSSION

In a series of three experimental conditions, we investigated how contextual cues influence statistical word learning and learner confidence in learning environments that contain both 1:1 and 2:1 word–object mappings. Across three conditions, we found that participants were able to acquire both 1:1 and 2:1 mappings at above chance levels in the retrospective tests, and that performance on these two types of mappings did not differ statistically. While contextual cues did not impact the overall level of performance on the retrospective task, they did exert an influence on the confidence ratings reported by learners during familiarization. Confidence ratings for both 1:1 and 2:1 mappings correlated positively with accuracy on the retrospective test completed at the end of familiarization. Notably, learners’ confidence in 2:1 mappings in the contextual cue conditions (i.e., gender and explicit cues) was significantly higher relative to the baseline (no cue) condition. This effect of context was not found for the 1:1 mappings. Overall, our findings suggest that the conscious awareness of learning for new mappings was stronger in the presence of a contextual cue marking the change between first and second familiarization than when new mappings were presented without any indication of the shift. Interestingly, the boost in confidence scores in the contextual cue condition was evidenced despite similar performance on the retrospective tests across conditions. This suggests that contextual cues may not only influence implicit statistical learning (e.g., Gebhart et al., 2009; Weiss et al., 2009), but also the interface between implicit processes and conscious awareness of learning, as indexed by the conscious appraisal of learning.

In a previous cross-situational word learning study (Poepsel et al., 2012), we demonstrated that the learning of 2:1 mappings was facilitated by adding contextual cues that distinguished between the two familiarization periods (e.g., a gender cue). Learning of 2:1 mappings was significantly greater in these conditions relative to a baseline condition containing no contextual cues. In the present experiment, we did not find an influence of contextual cues on the learning of many-to-one mappings as indexed by performance on retrospective tests. This may have been due to methodological differences between the studies. In the current experiment, the evidence for the second mapping was unambiguous (i.e., there was a 1:1 correspondence in the second familiarization period between the object and its new label), whereas in the second familiarization of our previous study (Poepsel et al., 2012) as well as that of Ichinco et al. (2009), the evidence for 2:1 mappings was more ambiguous. The difference in findings between studies suggests that contextual cues may not facilitate statistical learning of multiple mappings when the input strongly suggests the presence of the second mapping. A similar result was reported by Yurovsky and Yu (2008) who found no differences in performance on retrospective tests when the input during familiarization was organized in a similar fashion to the present study. Kachergis et al. (2009) point out that these differences in methodology and the variance in learner’s adherence to mutual exclusivity may be best understood within the framework of traditional associative learning. Learners can come to disregard the bias toward mutual exclusivity provided they have sufficient evidence for a new mapping. Without this evidence (as in the case of Ichinco et al., 2009), the learning of a new mapping remains effectively blocked (see Kachergis et al., 2009). However, we note this framework cannot explain why we did not find a primacy preference when the first and second objects were presented together in a preference test whereas Yurovsky and Yu (2008) did, given that both experiments contained equivalent evidence for the new mapping. Future efforts will endeavor to better understand this discrepancy by presenting the preference test before testing the new mappings to rule out the possibility that having learners identify the label just after the second familiarization (and prior to the preference test) did not inadvertently increase the preference for the recency mapping.

As in our previous experiment (Poepsel et al., 2012), we also found that performance on 1:1 mappings in the first familiarization was significantly higher than performance on 1:1 mappings in the second familiarization. An experiment with a similar paradigm by Ichinco et al. (2009) did not report similar findings as their participants exhibited relatively equal performance on 1:1 mappings between familiarizations. While we cannot account for this discrepancy between this study and the two studies conducted in our lab, we speculate that familiarity with the transferred first familiarization objects may have interfered with learning new associations for the second familiarization objects. While performance was above chance in the second familiarization, it is evident that the task of acquiring a large number of word–object mappings across multiple familiarization phases was taxing for learners.

Several recent cross-situational statistical learning studies have investigated the link between knowledge states in the moment of learning and performance on retrospective tests. For instance, Vlach and Sandhofer (2014) found a relationship between retrieval dynamics (i.e., the ease or difficulty of retrieving information during learning) and later retention of mappings in a cross-situational task. Specifically, initial difficulty in mapping retrieval during training predicted greater levels of mapping retention at a later test. A study by Medina et al. (2011) noted that the point in training at which disambiguating information about a mapping is received influences acquisition of that mapping. Thus, an earlier introduction of disambiguating information facilitated acquisition of a mapping, while a later introduction was not predictive of learning. In the present study, we hypothesized that contextual cues during familiarization to multiple mappings would facilitate remapping (i.e., that the contextual manipulation would disfavor adherence to mutual exclusivity during online learning). While contextual cues during familiarization did not exert an influence on the level of performance as measured by retrospective tests, they did influence the conscious appraisal of learning. Specifically, we found that learners in the contextual cue conditions were significantly more aware of learning the 2:1 mappings than were subjects who did not have an explicit cue. Without the contextual cue corresponding to a shift in structures, participants were not aware of having acquired the second mapping (though, notably, participants in all conditions were equally aware of having acquired the 1:1 mappings). The confidence ratings themselves correlated with performance on the retrospective tests, suggesting that this measure was an accurate index of awareness of learning. Dienes and Scott (2005) have asserted that a positive correlation between confidence ratings and accuracy indicates that knowledge is available to conscious manipulation, (but see also Hamrick and Rebuschat, 2012). While prior studies have demonstrated that contextual cues that correspond to changes in structure can influence implicit measures of statistical learning (Gebhart et al., 2009; Weiss et al., 2009; Mitchel and Weiss, 2010), here we have demonstrated that contextual cues can also influence awareness of learning.

Overall, our findings accord well with the notion that statistical learning can result in both implicit and explicit knowledge (e.g., Hamrick and Rebuschat, 2012). While some have described statistical learning as primarily an implicit process (e.g., Conway and Christiansen, 2006), there are several studies suggesting that the output of statistical learning may also be comprised of an explicit component. For example, Franco et al. (2011) used a process-dissociation procedure (PDP) to determine whether the representations formed in a speech segmentation task were available to conscious manipulation. During training, participants were exposed to two artificial languages sequentially, which were differentiated by a contextual cue (i.e., a voice change) as well as a brief pause. In the PDP, learners engaged in two tasks: an inclusion task, in which an auditory stimulus was judged as having been encountered in the training or not; and an exclusion task, in which a stimulus was categorized as belonging to either the first or second artificial language. While implicit knowledge of structure can support success on the inclusion task, only explicit knowledge can explain success in the exclusion task, as simple familiarity with learned structures may impair a learner’s ability to determine from which of several inputs a particular structure arises. Franco et al. (2011) found that learners acquired both artificial languages and performed above chance on the inclusion and exclusion tasks, suggesting that the knowledge acquired via statistical learning involves both an implicit and explicit component. This conclusion was also reached by Hamrick and Rebuschat (2012) who discovered that learners perform better in an intentional cross-situational word learning paradigm than they do in incidental learning conditions, as measured by performance on confidence ratings and source attributions.

Given the evidence that learners may be aware of the structures they acquire using a statistical learning mechanism, what is the specific contribution of contextual cues to learning? In environments presenting multiple inputs to learners, contextual cues may facilitate rapid discrimination of structures that arise from distinct inputs (as in the exclusion task of the PDP discussed above). Gebhart et al. (2009), for instance, found that when two artificial languages were presented sequentially, in the same voice, and for equal durations, learners acquired only the first language. When the duration of the second language was tripled relative to the first, learning of both languages followed; however, an essential task of any learner is to quickly detect changes in the learning environment, and from there to decide whether to incorporate those changes into an existing representation, or accommodate them with a new representation (Qian et al., 2012). Thus, when the languages were distinguished by a contextual cue (e.g., a voice change or an explicit cue) learners acquired both languages with equal exposure to each. Arguably, such highly salient contextual cues reduce uncertainty regarding points of transition between inputs, and so may serve as shortcuts for learners faced with the challenge of acquiring multiple inputs. Specifically, contextual cues seem to refocus a learner’s attention on the structures available in the input, such that a learner may quickly determine whether the structures match those of a previous input or arise from a new distribution. As a number of recent results indicate that attention is necessary for both auditory and visual statistical learning (e.g., Toro et al., 2005; Turk-Browne et al., 2005), the suggestion that contextual cues exert their influence on learning by redirecting attention to features of the input undergoing change seems highly plausible.

In sum, our findings support the suggestion that contextual cues impact the acquisition of multiple inputs, in this case how learners form 2:1 mappings in a CSSL paradigm. We further posit contextual cues (such as changes in speaker voice) likely help direct the learner’s attention to changed features of the input. In previous studies, this has been evidenced by improved performance in multi-stream segmentation tasks (e.g., Gebhart et al., 2009; Weiss et al., 2009) or multiple mappings in CSSL (Poepsel et al., 2012). In this study, despite stable performance in the retrospective tests of learning (likely a function of the type of evidence provided for multiple mappings), we found that learners were nevertheless more aware of their learning when provided with a contextual cue. This suggests that contextual cues to change may result in a more nuanced effect on learning, even without concomitant gains in implicit learning.

## Conflict of Interest Statement

The authors declare that the research was conducted in the absence of any commercial or financial relationships that could be construed as a potential conflict of interest.
